# Approaching Adversarial Example Classification with Chaos Theory

**DOI:** 10.3390/e22111201

**Published:** 2020-10-24

**Authors:** Anibal Pedraza, Oscar Deniz, Gloria Bueno

**Affiliations:** VISILAB, University of Castilla La Mancha, 13001 Ciudad Real, Spain; oscar.deniz@uclm.es (O.D.); gloria.bueno@uclm.es (G.B.)

**Keywords:** adversarial examples, entropy, Lyapunov, chaos theory, deep learning

## Abstract

Adversarial examples are one of the most intriguing topics in modern deep learning. Imperceptible perturbations to the input can fool robust models. In relation to this problem, attack and defense methods are being developed almost on a daily basis. In parallel, efforts are being made to simply pointing out when an input image is an adversarial example. This can help prevent potential issues, as the failure cases are easily recognizable by humans. The proposal in this work is to study how chaos theory methods can help distinguish adversarial examples from regular images. Our work is based on the assumption that deep networks behave as chaotic systems, and adversarial examples are the main manifestation of it (in the sense that a slight input variation produces a totally different output). In our experiments, we show that the Lyapunov exponents (an established measure of chaoticity), which have been recently proposed for classification of adversarial examples, are not robust to image processing transformations that alter image entropy. Furthermore, we show that entropy can complement Lyapunov exponents in such a way that the discriminating power is significantly enhanced. The proposed method achieves 65% to 100% accuracy detecting adversarials with a wide range of attacks (for example: CW, PGD, Spatial, HopSkip) for the MNIST dataset, with similar results when entropy-changing image processing methods (such as Equalization, Speckle and Gaussian noise) are applied. This is also corroborated with two other datasets, Fashion-MNIST and CIFAR 19. These results indicate that classifiers can enhance their robustness against the adversarial phenomenon, being applied in a wide variety of conditions that potentially matches real world cases and also other threatening scenarios.

## 1. Introduction

**Figure 1:**
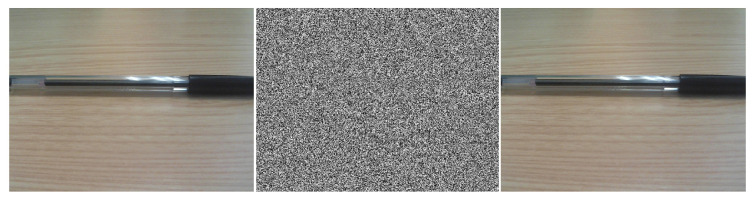
Adversarial example. From left to right: original image (classified as “ballpoint”), noise added, resulting adversarial image (classified as “speedboat”).

Adversarial examples are a hot topic in current machine learning research. They consist of slight modifications of a neural network input (barely noticeable by human perception) which cause huge differences in its output. When applied to images, for example, small perturbations in carefully selected pixels can cause a classifier to predict a different class, even when the original and the perturbed image remain practically indistinguishable to human observers [1]. For example, Figure 1 shows an image which, with a small perturbation, is classified as a speedboat rather than a ballpoint.

This phenomenon has strong implications in terms of security, as more and more systems are relying on artificial intelligence. There is the risk that models can exhibit such absurd flaws in real life, with serious implications in their effective utility.

This threat is even more relevant due to the topics on which the deep learning methodology is applied. Since it was first proposed in [2], the evolution of the field has increasingly grown. In that seminal work, the main concepts of convolution and pooling layers, their interaction and effects on the results, along with the parameters involved in the training procedure were described and analyzed. This first work was applied to a complex task, i.e., real-world image classification on ImageNet (more than a million images with 1000 different categories). From that moment on, the research and application of continuously evolving, increasingly complex, convolutional neural networks has grown exponentially. One of the most interesting fields of application is healthcare. In this respect, huge efforts have been made to advance in deep learning applied to medicine. For example [3], covers this topic, with interesting insights about the main contributions in tomography, resonance image analysis or even cancer assessment. Regarding industry, deep learning has also been applied to quality control of manufacturing processes. For example [4], covers the application of fruit quality evaluation for food factories. Finally, another interesting field of application is robotics, which is in a close relationship with computer vision. A combination of both could potentially build automated robots with the capability of full environment understanding. For example [5], considers the main advances in 3D data interpretation algorithms. This is interesting for a wide variety of applications such as automated reconstruction of building diagrams or environment-aware robots to perform automated tasks both in indoor or outdoor conditions. Given this context of widespread application of the methodology in our society, it is understandable the need for research in threatening phenomena such as adversarial examples.

The literature available on adversarial examples has grown significantly in the past few years. In parallel with the so-called attack methods (algorithms to generate adversarial examples) several defense methods have been proposed which intend to build more robust networks. This can be achieved, for example, by performing more training stages with adversarial examples [6], or by modifying the weights that the network learns during training [7]. Other methods aim at performing a pre-classification of a potential adversarial input before it is fed into the network [8].

We notice that deep convolutional networks can be considered chaotic systems. Adversarial examples represent tiny changes in the input that produce wildly different outputs. In the context of chaos theory, the Lyapunov exponents are a set of numerical values that measure how chaotic a system is [9]. The largest one is usually known as the Maximal Lyapunov exponent (MLE) and it determines a notion of predictability for the dynamical system. In our case, the MLE (the first one) and other three subsequent exponents would be extracted.

Lyapunov exponents cannot be calculated analytically, so different estimation algorithms are available. In the algorithm of Eckmann et al. [9], the one used in our work, the input is a flattened version of the original image matrix, with a total of 784 values vector (since images have 28 × 28 pixel size). After that, a Jacobian matrix is estimated and the Lyapunov exponents are obtained from its eigenvalues.

The spectrum of Lyapunov exponents can be defined as in Equation (1), being the first so-called MLE.

(1) $$\left\{\lambda_{1},\lambda_{2},\cdots ,\lambda_{n}\right\}$$

The Lyapunov exponents describe the behavior of vectors (here, the image) in the tangent space of the phase space and are defined from the Jacobian matrix defined in Equation (2).

(2) $$J^{t}\left(x_{0}\right)=\frac{df^{t}\left(x\right)}{dx}|x_{0}$$

This Jacobian defines the evolution of the tangent vectors, given by the matrix *Y*Y, via Equation (3).

(3) $$\dot{Y}=JY$$

The preliminary condition is that, on the initial point Y(0), the distance between different vectors in the space is infinitesimal. However, if the system is chaotic, the matrix *Y* describes how a small change at the point x(0) propagates to the final point $x_{\left(t\right)}$. The limit described in Equation (4) defines a matrix Λ. Then, the Lyapunov exponents $\lambda_{i}$ are defined by the eigenvalues of Λ.

(4) $$\Lambda =\lim_{t\to \infty }\frac{1}{2t}log\left(Y\left(t\right)Y^{T}\left(t\right)\right)$$

Given that each $\Lambda_{i}$ is an eigenvalue of Λ, the corresponding Lyapunov exponent $\lambda_{i}$ is obtained as defined in Equation (5). Usually, a single positive value is indicative enough for the presence of chaos in the system. To ensure all possible positive exponents are calculated, the first four exponents are estimated from any image.

(5) $$\lambda_{i}\left(x_{0}\right)=log\Lambda_{i}\left(x_{0}\right)$$

Despite the appealing analogy with chaotic systems, few works have leveraged chaos theory in the context of adversarial examples. The seminal work [10] used the Lyapunov exponents extracted from the flattened input image to classify it as legitimate or adversarial example. In that work, the authors showed that adversarial examples produce consistently higher values of the first Lyapunov exponents, which allowed for a good classification between legit images and adversarial images.

Chaoticity and entropy are known to be related. For example [11], states that the relationship between the Maximum Lyapunov Exponent and the ‘permutation entropy’ has been observed both analytically and experimentally. Lyapunov exponents are also related with Kolmogorov-Sinai entropy (KSE) and Shannon entropy through Pesin’s theorem, see [12]. The theorem states that the KSE is equivalent to the sum of the positive Lyapunov exponents. Moreover, the same author formalizes in [13] the connection of KSE as a measure of randomness and Shannon entropy as a metric for disorder. Following the same line of research, the work in [14] also relates the fields of dynamical systems and information theory. In this case, chaoticity in hidden Markov models can be described with Shannon entropy. Finally [15], employs both entropy and Lyapunov exponents to study the chaos in the architecture of a layered deep neural network.

The connection exposed above suggests that entropy-changing image transforms (such as a simple histogram equalization) may have an effect on an adversarial classification system that is based on Lyapunov exponents. The experimentation performed in the aforementioned paper [10] tested adversarial classification using a number of well-known adversarial attack methods. However, it did not consider the effect of other kinds of image noise in the robustness of the method, particularly those that may change image entropy. This is precisely the hypothesis that we test in this work. Based on our results, we further propose entropy itself as a useful feature to classify adversarial examples from legit images.

In [16], an entropy based adversarial classification method was proposed. Saliency maps are computed over a three-channel input in the network gradients for a backward computation through the network. These maps are useful to visualize the pixels that contribute the most in the classifier decision. In legit images, they highlight the most discriminant regions of the images, like edges and important features. However, adversarial saliency maps are less sparse, with more spatially distributed highlighted areas. When entropy is computed in these single channel maps, values are a bit higher in adversarial images since more information needs to be codified. However their experimentation is not conclusive, as only one adversarial method with few parameters is tested, and entropy values do not differ much between both classes. In our experimentation, entropy is calculated over the raw images, so the information of the perturbations is kept with more fidelity and entropy values have greater difference when calculated over legit and adversarial images. Moreover, a comprehensive collection of adversarial attacks and images processing methods are tested to obtain significant results.

Regarding similar adversarial classification methods in the state of the art, there are some interesting proposals to be discussed. In [17], the image space of legit and adversarial images are compared, along with the predicted class, to check whether the image has been potentially perturbed. The obtained results are useful with a 99% accuracy in detecting adversarials with different levels of perturbations, but only raw perturbations are studied and only the PGD attack method is tested. Using the internal parameters and layer activations to detect adversarial examples [18], provides an interesting study that achieves a certifiable detection rate under certain restrictions in the $L_{\infty }$ norm. Five different common attacks are tested, although the method is able to achieve good results only on the MNIST dataset. In [19], an autoencoder is built for detection, where the reconstruction of the candidate image determines the probability of an adversarial origin. Moreover, the approach is reused to build an adversarial robust network with up to 76% accuracy for the Sparse attack. Similar to [17], the work proposed in [20] studies the image data to classify an image into a potential adversarial. In this case, through feature alignment with an external model, the method is able to detect the adversarial image and fix the classifier decision. However, the employed dataset is a customized version of PASCAL VOC, ImageNet and scraped images from the internet, which results in a difficult framework for comparison. Finally, a comparable approach to ours is given by [21], where statistical analysis is used to predict whether an image behaves as an adversarial or not. Gaussian noise is compared to regular adversarial attacks to check the validity of the method. However, the studied attacks do not include the latest and more powerful proposals in the state of the art.

In order to compare the different features of the aforementioned methods, Table 1 shows a comparison of their approaches, studied datasets, and the tested attacks or methods.

This paper is organized as follows: Section 2 describes the dataset and specific adversarial methods that are used in this work. Section 3 provides a comprehensive description of the experiments that have been carried out, including Lyapunov exponents for adversarial classification and the proposal of entropy for the same purpose, as well as a comparison of both methods. Finally, Section 4 discusses the main results obtained, providing also an insight on relevant topics for future work.

## 2. Materials and Methods

**Figure 2:**
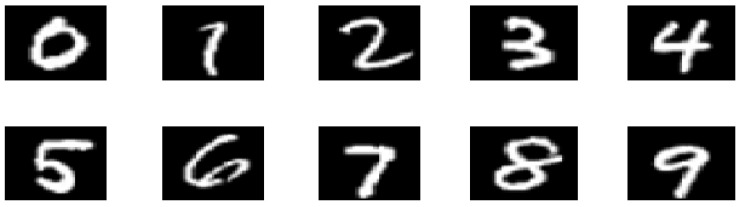
Examples of MNIST dataset.

In this work, we employed three datasets to compare the method on a wide range of images and conditions. First, the well-known MNIST dataset [22], which contains handwritten digits with 10 different classes (from 0 to 9) and size 28 × 28 (grayscale single channel images), as shown in Figure 2. The dataset has 60,000 train images and 10,000 test images. It is the same dataset used in the reference paper [10] and one of the most employed datasets in adversarial example research. The reason is that the images are simple enough to study the perturbations in detail.

**Figure 3:**
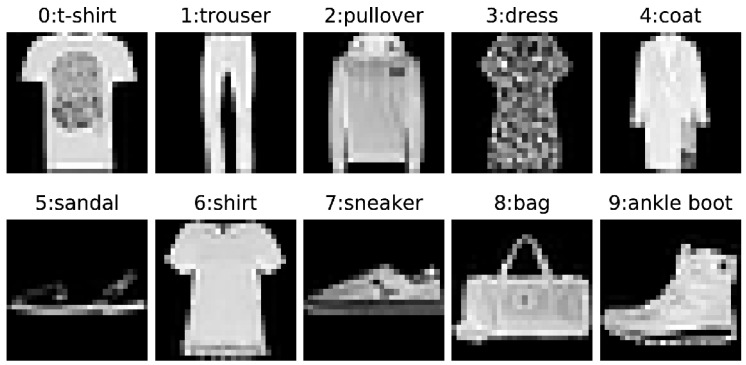
Examples of Fashion-MNIST dataset.

This work also used the Fashion-MNIST dataset. It was developed by the Zalando company to improve research in image processing [23]. Inspired by the MNIST dataset, it contains 10 classes of popular kinds of clothing articles instead of digits, with 60,000 training and 10,000 test images. The structure is the same, with 28 × 28 single channel images. A sample of this dataset is shown in Figure 3.

**Figure 4:**
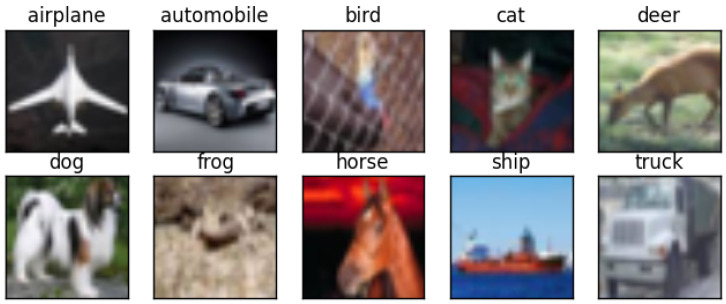
Examples of CIFAR dataset.

Finally, a third widely used dataset was employed in this work. CIFAR-10 was developed as a subset of miniatures from the ImageNet dataset [24], to make research available with less computational power and less time-consuming processes. It contains 32 × 32 color images of common objects, such as birds, ships or airplanes. This dataset contains 50,000 images for training and 10,000 images for test. Figure 4 shows a sample for the different classes. For the purpose of this work the grayscale variant of the dataset was employed.

To craft the adversarial examples from these datasets, the target model is a LeNet architecture [25], one of the most common architectures in adversarial research. It was used because it has enough parameters so adversarial images are also considered adversarials by other more complex networks [26], in what is called adversarial transferability. Furthermore, it has enough input size for the datasets employed in this work while maintaining an affordable and bounded computation complexity for Lyapunov exponents.

In order to cover a wide range of adversarial attacks, twelve different attack methods have been tested, from the most common in adversarial research comparisons to the latest contributions on the topic. These have been proposed during the recent years in which adversarial example research has grown significantly.

There are two main approaches in adversarial attacks. The first one is the so-called white box, in which the algorithm has full access to the data and internal parameters of the model under threat. The other approach is called black box. In this case, the algorithm crafts the adversarials estimating the model behaviour with different techniques. For example, by querying the model and observing its response to different perturbations, or by training a similar model to the original to develop the adversarials.

In the publication that serves as the reference for this study [10], five of the most common attacks are employed, which are included in this work for reproducibility and comparison purposes. In the following, those attacks are described.

One of the most widely used attacks is Carlini and Wagner (CW), the attack proposed in [27]. It defines a cost function to modify the input of the network, in which the distance to the original sample has to be minimized at the same time as the perturbations are introduced. As a result, the examples crafted with this method are much closer to the original inputs, and, in consequence, they are more difficult to be detected visually or through defense techniques like defensive distillation in [28]. As one the first steps in adversarial attacks, Fast Gradient Sign Method (FGSM) was introduced by [29]. This method was one of the first to show the phenomenon of adversarial examples in deep neural networks. They proposed a methodology which calculates the gradient of the cost function with respect to the input of the network. Using this information, this method computes a single perturbation to the image to maximize the resulting gradient in the direction of a class different than the groundtruth. As a variant of this attack, the Madry [30] method applies some restrictions for the random initialization of the datapoint seed are fixed. The different values for the initial conditions in the algorithm can lead to more robust adversarials, depending on the network complexity. Finally, the Jacobian Saliency Map Attack (JSMA), which was introduced in [31], is a method that takes advantage of the features that have more impact in network decision. Building the saliency maps, the method discovers the key pixels that have a significant impact in the decision when perturbed, so it can minimize the number of pixels and amount of perturbations to perform the adversarial attack.

In this work, a wider range of attack methods is considered. Variants of the FGSM and Madry methods are tested. They have been developed to make the approach even more robust or adaptive. For example, the Basic Iterative Method (BIM), as described in [32], was a revision that performs the gradient estimation iteratively, so the adversarial example is built using several small steps. For this reason, the crafted perturbations are more effective with less perturbation in comparison. Projected Gradient Descent (PGD) as described in [30], is a further step variant of the Basic Iterative Method [32]. In this attack, after each iteration, the selected perturbation is projected on a theoretical Lp-ball (which uses a selected distance, being **L_0_**, *L_2_* or Linf, for example) of specified radius, thus keeping the perturbation small and in the range of the input data. As the top and most recent step in this family of attacks, the Sparse-L1 Descent (SL1D) [33] computes their perturbations in a projection over the L1 distance. This has significant advantages in terms of adversarial robustness, since images are less perturbed (and therefore less detectable).

Regarding more classic approaches, DeepFool was one of the first attack methods to successfully craft adversarials in large scale datasets (such as ImageNet), as exposed in [34]. It estimates the theoretical plane for perturbations within a boundary in which the classifier remains predicting the same class, in order to overcome this frontier and calculate the necessary perturbations to produce the adversarial example. Coming from a defensive approach, Virtual Adversarial (Virtual), proposed in [35], applies a specific algorithm extracted from adversarial training, to produce the adversarials even without the information of the output labels, only with the gradients and parameters. For this purpose, it was one of the most successful methods to take advantage from adversarial training to craft better adversarials. With a different approach, Elastic-Net Attack (EAD), described in [36], proposes a regularization optimization problem to compute small L1 distances (which can be extended to the *L_2_* domain). In comparison to other methods, it is able to break some popular defenses such as distillation and adversarial training, although the general performance is similar to other methods such as C&W.

Finally, regarding the black box paradigm, we first consider the Spatial Transformations method (Spatial) [37]. Without any knowledge about the internal parameters of the model, this method computes slight spatial transformations such as translations and rotations, checking whether the output class changes or not, to iterative build the adversarial examples. The most recent approach in this paradigm is the HopSkipJump attack [38] (previously called Boundary Attack). Only by querying the model with slight perturbations, this algorithms is able to estimate the responses of the model to new perturbations and compute them efficiently.

The aforementioned attacks have been applied with the specific parameters that are detailed in Table 2. Most of them are common to several attacks, such as the number of iterations of the maximum perturbation allowed (usually so-called epsilon). These are the most important to control the quality of the adversarial examples. For this reason, they have been set to the default values proposed by their respective authors in each referenced publication. Therefore, these configurations are proved to be the most suitable to perform as robust as possible adversarials, with optimal computation time. If recommended values were not employed, the attacks may produce over perturbed images, which would lead to sub-optimal adversarials. Moreover, increasing the number of iterations or search steps would increase the processing time exponentially, with marginal benefits. For example, axis and angle rotation limits in the Spatial attack have been chosen to stay in a visually acceptable range (over rotated numbers or objects may look suspicious for humans). Other parameters, such as gamma/xi/tradeoff, common in several attacks, are used as corrective factors of the algorithms and they have not substantial influence on the final results. Finally, secondary parameters, not mentioned in the detailed table, are used to adapt the adversarials to each specific dataset. Usually they are employed to clip and bound the resulting adversarials to the representation range of the images. These are not relevant and constitute low-level implementation details.

On the other hand, image processing methods can produce noise and perturb an image, in some cases even producing missclasifications. In this respect, an optimal adversarial discriminant method should be robust to those, neglecting the effect of those other sources of noise when they do not affect class decision, and pointing the corresponding adversarials when the model is fooled. In this work, these image processing transforms are performed over both clean (legit) test and adversarial images. The objective is to check whether the Lyapunov-exponents method for detecting adversarial images is robust to such simple sources of noise. That is, we intend to analyse failure rates when confronted with such simple image transformations. Such robustness assessment was also done in [10]. In this respect, if the method classifies a (transformed) legit test image as an adversarial, then this legit image will be rejected by the system, which must be considered an error. Similarly, if a (transformed) adversarial image is no longer detected by the method, that is also an error. Another purpose of the image processing is to modify the entropy for both legit test images and adversarial examples. This also allows to check the robustness of the Lyapunov method, since we know that entropy and Lyapunov exponents are somehow related [11]. That is, with this we want to detect cases in which entropy-altering transformations (that do not change class) make the method classify legit images as adversarials and vice versa.

Regarding the collection of image processing methods, hereinafter the ones employed in this work are explained. Histogram Equalization (EQ) is usually employed to enhance image contrast. In EQ, the image intensity histogram is transformed to be equally distributed over the intensity range. As a variant of this method, Contrast Limited Adaptive Histogram Equalization (CLAHE) applies a rule in which the maximum value of the histogram for a pixel is clipped in relationship with the the values of its vicinity. Other methods, such as Gaussian filtering, apply a kernel over the image. The filtering is performed with a normally distributed unidimensional convolution, with a more or less intense blurring effect depending on the parameters. In our experiments, the standard deviation of the kernel distribution is 1 (sigma parameter), and the order of magnitude is 0. With these values, the result is partially diffused without making the object unrecognizable. Finally, there are methods that introduce more random effects. Gaussian noise performs a distributed additive noise that follows the normal distribution. It can be applied over the whole image, according to the variance of vicinity at each pixel. Another example is Poisson noise (Poisson distributed additive noise), which is only defined for positive integers. To apply this noise type, the number of unique values in the image is found and the next power of two is used to scale up the floating-point result, after which it is scaled back down to the floating-point image range. Other kinds of noise perform simpler operations, such as ‘pepper’ noise (random pixels to 0), ‘salt’ noise (random pixels to 1), ‘salt & pepper’ noise (random pixels to 0 or 1). Finally, ‘speckle’ noise is produced by a multiplicative factor (*n* × image). In this case, to preserve the image integrity the “n” factor is chosen to have 0 mean and 0.01 variance.

Again, the objective is to alter the adversarial examples with these transformations and check if the detector can still distinguish between legit and adversarial images.

**Figure 5:**
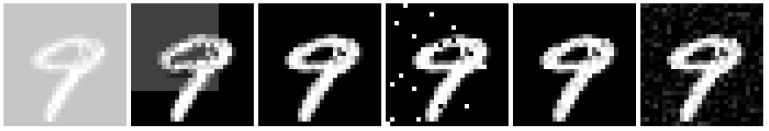
Examples of noise and processing (on a CW attack) in the MNIST dataset. From left to right, top to bottom: EQ, CLAHE, Poisson, salt and pepper, speckle and local variance Gaussian noise.

**Figure 6:**
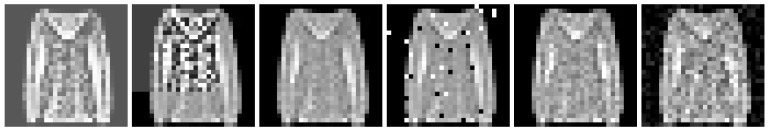
Examples of noise and processing (on a CW attack) in Fashion-MNIST dataset. From left to right, top to bottom: EQ, CLAHE, Poisson, salt and pepper, speckle and local variance Gaussian noise.

**Figure 7:**
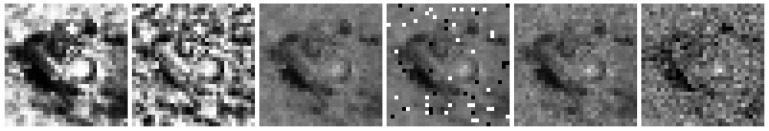
Examples of noise and processing (on a CW attack) in the CIFAR dataset. From left to right, top to bottom: EQ, CLAHE, Poisson, salt and pepper, speckle and local variance Gaussian noise.

A good adversarial classifier should be robust to different sources of noise, such as those described above and shown in Figure 5. Regarding the other datasets, Figure 6 shows the examples for CW attack in the Fashion-MNIST dataset and Figure 7 shows the same methods for a sample in the CIFAR dataset.

## 3. Experiments

In order to compare the original methods proposed in the reference publication and the claims of our approach, several experiments have been proposed. First, all the attacks and processing methods described in the previous sections are compared in the task of using the Lyapunov exponents to classify between non adversarial (legit test images) and adversarially perturbed images (adversarial examples, obtained by attacking the legit test images). For this purpose, a state of the art LeNet-like architecture (i.e., an end-to-end deep network) is used for all the adversarial related processes (testing of datasets, adversarial crafting with the different attacks, etc). In consequence, the method is able to obtain the best generalizable model possible, therefore reducing the impact of overfitting in the results. Then, using the test set of each dataset, a random subset of 100 images are taken to craft the adversarials and apply the processings, in each experimental set. This is applied in a 10-fold cross validation procedure, so the results are shown as the average of the whole set of runs. Finally, in each execution, a classic machine learning method is employed to classify the features (Lyapunov exponents, entropy values, ...) extracted from the images, as a simple linear classification problem of 1, 4 or 5 features (if entropy, Lyapunov exponents, or both are used) to characterize each sample, and 2 classes: adversarial or legit image. The employed method is a Support Vector Machine (SVM) classifier, since it is one of the most powerful and optimized methods for this kind of task (linearly classifying a small set of features). This decision is in line with other works [10]. Regarding other options, no further benefits have been observed (neither accuracy nor computation time) in the initial tests by using different classifiers from the supervised machine learning family, such as linear discriminant, k-nearest neighbors or boosted trees. For the kind of data employed in this work, SVM is powerful enough to obtain the maximum accuracy with the available features. The specific parameters to obtain the best results from the method are the following: Gaussian kernel function (as the most suitable to fit the data in only two classes), 0.5 kernel scale and standardization applied to normalize the values in a closer range (which helps the Gaussian function to fit the data) and a box constrain of 1 to fix the number of support vectors per class. As a summary of this experimentation, Table 3, Table 4 and Table 5 show, for the three datasets, the classification accuracy. In the first column, we can observe the “base” adversarial images, with no processing method applied (i.e., the raw adversarial perturbed images). The rest of columns show the accuracy when adversarial images are further processed with the corresponding methods.

**Figure 8:**
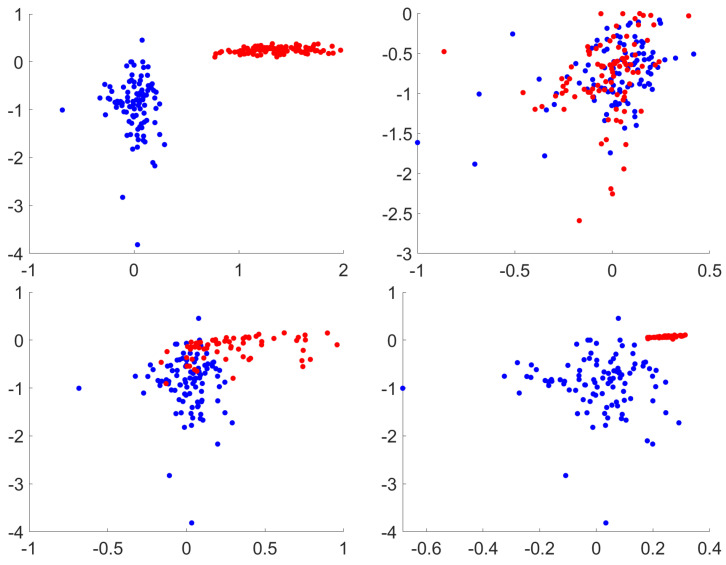
Lyapunov classification for CW attack. From left to right, top to bottom: no processing, EQ, CLAHE and Gaussian local variance noise. Only first and second exponents are represented. Test in blue, adversarials in red. Best viewed in color.

In general, all of the image processing operations have a detrimental effect in the adversarial example classification method. We illustrate this in the case of MNIST. Figure 8 shows the first two Lyapunov exponents for some of the processing methods in the CW attack in this dataset. As it can be observed, the classification is more accurate when no processing is applied, since the test images (blue points) are better separated from the adversarial images (red dots). This is mainly because the values for the first exponents are higher (more positive), and shifted to the right in the *x*-axis. Nevertheless, when image processing transformations are applied, such as equalization (normal or adaptive), adversarial images are no longer distinguished from test images so clearly. For this reason, the performance in classifications drops from 100% to 57.71% and 84.85%, respectively. Finally, local variance Gaussian noise does not show any impact on the Lyapunov exponents, and, in consequence, the classification of adversarial images remains with perfect score.

**Figure 9:**
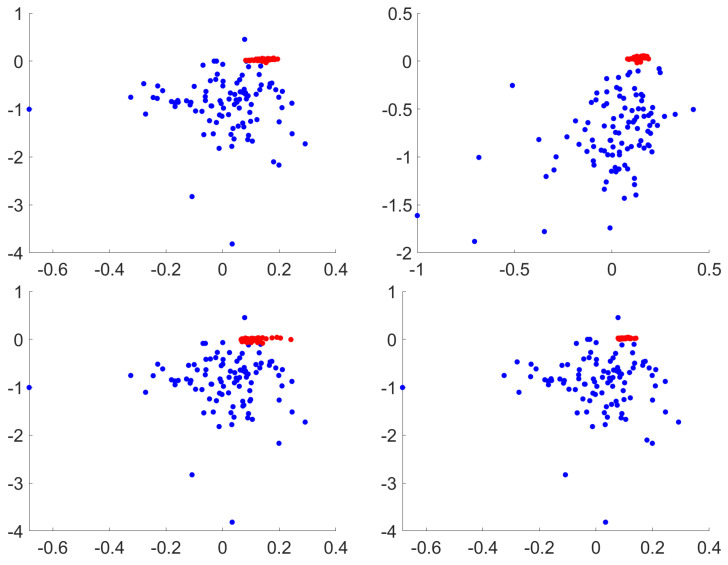
Lyapunov classification for PGD attack. From left to right, top to bottom: no processing, EQ, CLAHE and Gaussian local variance noise. Only first and second exponents are represented. Test in blue, adversarials in red. Best viewed in color.

**Figure 10:**
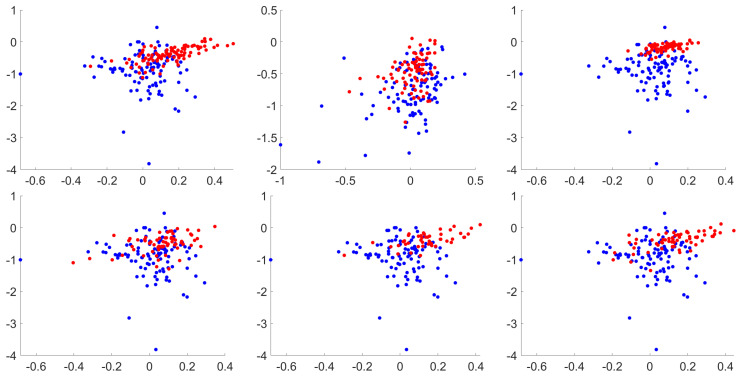
Lyapunov classification for EAD attack. From left to right, top to bottom: no processing, EQ, CLAHE, Poisson, salt and pepper and speckle noise. Only first and second exponents are represented. Test in blue, adversarials in red. Best viewed in color.

In contrast, for the PGD attack (Figure 9) it is observed that there are no major changes in the Lyapunov exponents distribution when image processing methods are applied, being that the reason to have near 100% accuracy for every single method for adversarial classification (as observed in the PGD row from Table 3). Themost recent black box attack also exhibit a good performance, ranging from 99% to 100% in all combinations. Finally, the attacks that reduce the Lyapunov accuracy the most are EAD and JSMA. The powerful Sparse attack is also affected, but with better values than the former attacks. A visualization of the Lyapunov exponents from test and adversarial images is shown in Figure 10. The adversarial images have, for the attacks considered, negative exponent values that are contained within the same region of the legit test images.

As it is observed in Table 3, Table 4 and Table 5, depending on the combination of attack and processing, the results can vary substantially. In consequence, we have conducted an additional investigation to explain the different behaviour depending on the attacks. As a result of this investigation, we have discovered that the *L_0_* metric (*L_0_* represents the total number of pixels that have been modified) shows a strong correlation with the accuracy of the Lyapunov exponents method. Table 6 shows the mean *L_0_* value for the base (unprocessed) adversarial images along with the accuracy obtained by Lyapunov exponents method.

Over a maximum *L_0_* value of 784 (since images are 28 × 28 pixel in size), we can see that when the method modifies less than a half of the pixels, the accuracy is penalized. Thus, it can be inferred that the Lyapunov exponents are very sensible to the number of pixels that are modified. Regarding the specified attacks FGM, PGD, BIM and Madry, those four adversarial attacks belong to the same family of algorithms, which may be the reason why they behave in a similar way, producing a similar kind of adversarials.

**Figure 11:**
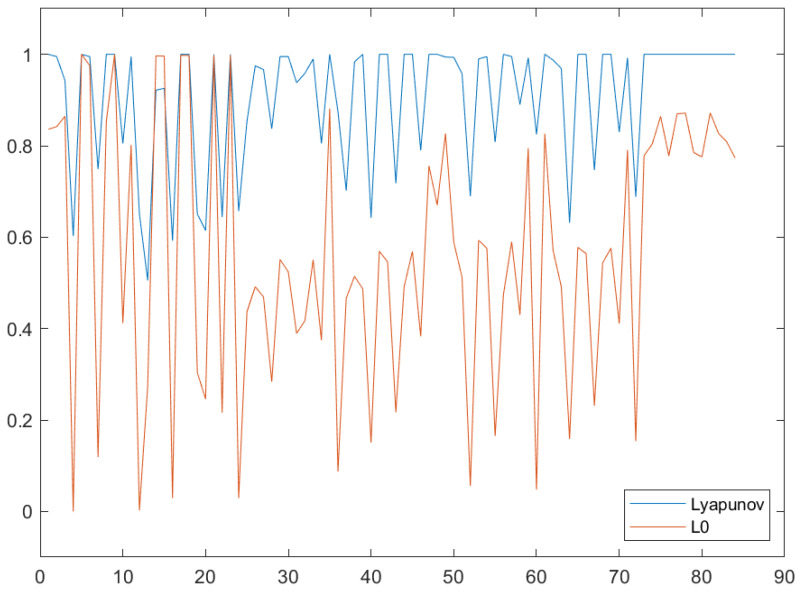
Correlation between Lyapunov exponents accuracy and $L_{0}$ metric. Best viewed in color.

When we extend the observation of *L_0_* values to the whole set of attacks and image processing methods in the MNIST dataset, the correlation is even more clear. In Figure 11, the 84 data points in the horizontal axis represent the total 84 different combinations of attack method and image processing effect. Regarding the vertical axis, note that the *L_0_* data has been normalized between 0 and 1 for visualization purposes. Furthermore, accuracies are presented in the 0–1 range too, for the same purpose. As it is observed, both follow the same trend, in which valleys on *L_0_* (less perturbed pixels) point to drops in accuracy and vice versa.

The same pattern is observed in the three datasets with a slight overall decrease in the accuracy for both the Fashion-MNIST and CIFAR datasets. The complexity of those datasets make it more difficult to classify the perturbations in the adversarial images. However, some combinations are still robust, such as Poisson, salt and pepper and speckle transformations for Fashion-MNIST, along with PGD, BIM and Madry attacks. Regarding the CIFAR dataset, PGD and particularly Spatial attacks remain with high performance in adversarial classification.

### 3.1. Effects of Entropy

Since entropy is known to be related to Lyapunov exponents [11], we propose to study how the aforementioned image processing methods affect the entropy of the images. Different image processing methods affect entropy differently so we first checked if the changes in entropy are correlated with the the accuracy of classification using Lyapunov exponents.

Entropy computes the quotient between frequency and probability of each possible intensity value to occur in the image range. Depending on the choice for the logarithmic base (*b*), the formula described in Equation (6) can be used to compute the Shannon entropy (base 2) [39], natural unit (base *e*) or Hartley (base 10).

(6) $$E=-\sum_{i}p_{i}\cdot log_{b}\;p_{i}\;,\;\;with\;b=2$$

In this expression, $p_{i}$ represents the probability of a pixel having the intensity value *i*. In this case, it ranges from 0 to 255 as the images are encoded with 8-bit single channel greyscale. These probabilities can be extracted from the image histogram. As a result, an image with a more compact histogram would have a lower Shannon entropy value and vice versa.

In Table 7, Table 8 and Table 9 the mean entropy of adversarial images is shown for the three datasets.

We can observe that images have a different “base” value depending on the attack method. When comparing each pair of test images (first row) and a given attack (the rest of the rows) it is observed that when the image processing produces an increasing entropy, Lyapunov exponents seem to classify the image with more accuracy. When the entropy of an adversarial attack row remain in the same average as the test row, it is more difficult to classify these images using the exponents. On the opposite, the processing methods that had their Lyapunov exponents on the same distribution of the base images, exhibit a marked decrease in their entropy values.

To further show that there is a relationship between both Lyapunov accuracy in adversarial prediction and entropy, we build a normalization method to exhibit empirically whether an increment in entropy has a correlation with better adversarial predictability with Lyapunov exponents. The aim of the normalization process is to point out that variations in entropy for the different attacks and processings (in comparison to the base (unaltered) images) are related to Lyapunov exponents accuracy. However, due to the different kinds of attacks and number of perturbations, the entropy for each attack range of values is very wide (between 2.75 and 9.3 in FGM, or 2.37 and 6.19 in EAD, for example). To compare them in relative terms, each row of entropy from Table 7 is normalized between 0 and 1. After that, each processing value for a given attack is subtracted from the corresponding base value. As a result, each resulting value represent the proportional increment/decrement in entropy for a given processing, with respect to the base images. As a result, we obtain Table 10.

Using this data, the correlation between the entropy data and the accuracy obtained with Lyapunov exponents has been studied. There are a total of 6 (methods) × 10 (attacks) = 60 different cases in which the accuracy of the Lyapunov exponents can be related with its corresponding increasing or decreasing mean value of the entropy for these images. Using the Pearson correlation index [40] with these pairs of data, the result shows a positive correlation of +0.43 and a *p*-value of 0.1 × 10${}^{-4}$. This shows that indeed there is a moderate (and statistically significant) correlation between the two quantities: when the entropy of the adversarial images is reduced, the accuracy of the Lyapunov exponents method is also lower.

The previous results lead us to check if entropy can be a feature that can be employed for adversarial prediction (instead of Lyapunov exponents). Following the same experimental conditions of the Lyapunov exponents, we extract the Shannon entropy of each image, as a single feature to determine whether an image is legit or adversarial (with or without any additional processing method). In this case the data has been classified using a linear discriminant analysis, which is enough for a single feature model. The results are shown in Table 11, Table 12 and Table 13 for the reference datasets.

The absolute difference of Lyapunov performance in Lyapunov exponent-based classification and Shannon entropy-based adversarial classification has been calculated in Table 14, Table 15 and Table 16.

Using the entropy as a feature to classify between legit and adversarial inputs for the MNIST datasets has some benefits but also some drawbacks. In total, 38% of the cases (32/84) increase the accuracy with respect to Lyapunov exponents, 24% perform the same (20/84) and 38% decrease the performance (32/84). So, at least, in 62% of the cases entropy is able to perform equal or better than Lyapunov exponents. Regarding the drawbacks, JSMA attack and localvar Gaussian show an even deeper negative impact, noting that this method is much more prominent to failure when perturbations are reduced in number and range (similar as stated for Lyapunov exponents in previous sections). Following the same pattern, the performance on the other two datasets is weaker, but still remarkable, considering than a single value/feature is used to characterize a legit or adversarial image, for such a wide variety of attacks and processings.

Furthermore, we have used the same format to compare between using both features vs using only Lyapunov exponents. The data was extracted from the comparison between Table 3 and Table 17, showing that in 52% (43/84) of cases the performance was improved, in 34% (29/84) it was equal and only in 14% (12/84) it was decreased. In this case, the correct statement is that in 86% of the cases the accuracy of the combination was equal or better than Lyapunov alone.

In comparison to MNIST, entropy is not so good at distinguishing between adversarial and legit images in Fashion-MNIST and CIFAR, since these datasets have similar entropy values in both kinds of images. As observed in Table 8 and Table 9 the entropy for the test images and the corresponding attacked ones have very similar values, so this feature by itself is not able to classify the two types of images with the same performance as Lyapunov exponents did.

The main reason is that backgrounds and objects are more defined in MNIST, so perturbations are better detected by the entropy. Fashion-MNIST and (especially) CIFAR have much more diffuse backgrounds and grey levels around and inside the object.

### 3.2. Lyapunov and Entropy Combined for Classification

In previous sections, Lyapunov exponents have been confirmed as a reliable method to classify adversarial images in most of the attacks and processing transforms. However, entropy also performed accurately for a significant amount of combinations in the three datasets, even though it was used as a single feature for discrimination. Although it did not perform as well in some datasets with specific methods, entropy shows to be a powerful and descriptive metric and provides useful information. For this reason, we decided to test the performance of a combination of both Lyapunov exponents and entropy, leading to a 5-feature classification problem (four Lyapunov exponents plus the Shannon entropy). To perform this task, a medium Gaussian kernel support vector machine classifier is employed, as in the raw exponents classification. The results are summarized in Table 17, Table 18 and Table 19 regarding the MNIST, Fashion-MNIST and CIFAR datasets, respectively.

**Figure 12:**
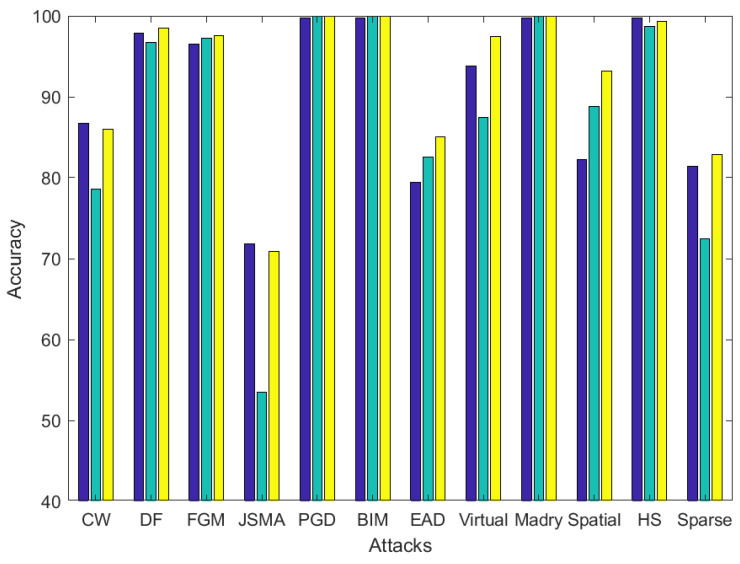
Accuracy comparison between using Lyapunov exponents (blue), entropy (green) and combination of them (yellow) in adversarial example classification on average for the image processing operations in the MNIST dataset. Best viewed in color.

**Figure 13:**
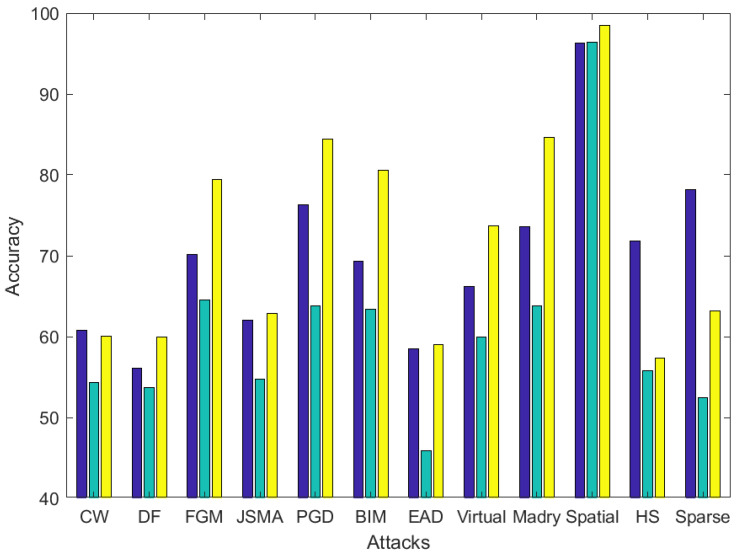
Accuracy comparison between using Lyapunov exponents (blue), entropy (green) and combination of them (yellow) in adversarial example classification on average for the image processing operations in the FASHION dataset. Best viewed in color.

**Figure 14:**
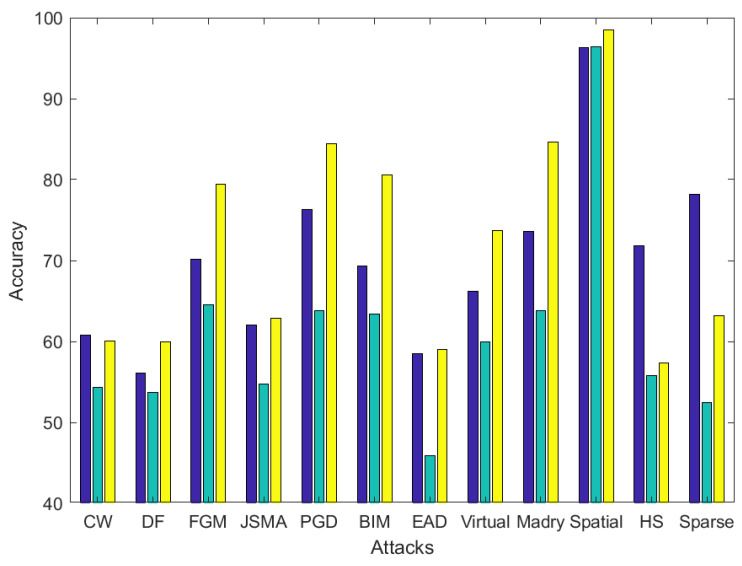
Accuracy comparison between using Lyapunov exponents (blue), entropy (green) and a combination of them (yellow) in adversarial example classification, on average for the image processing operations in the CIFAR dataset. Best viewed in color.

In order to quantify the variation of performance, the following Figure 12, Figure 13 and Figure 14 show the accuracy variation when Lyapunov exponents, entropy and both both are used to classify adversarial images. Each bar represents the mean accuracy of the image processing operations for the 10 different adversarial attacks.

In the MNIST dataset, as observed in Figure 12, an average of 2% accuracy increment is obtained: 91% for Lyapunov, 88% for entropy and 93% for the combination. The overall performance is increased significantly in two specific attacks, EAD and Spatial (around +14%).

In the Fashion-MNIST dataset, as observed in Figure 13, an average of 5% accuracy increment is obtained: 81% for Lyapunov, 74% for entropy and 86% for the combination. The overall performance is increased significantly in two specific attacks, DeepFool and EAD (around +8%).

In the CIFAR dataset, as observed in Figure 14, an average of 5% accuracy increment is obtained: 69% for Lyapunov, 62% for entropy and 74% for the combination. The overall performance is increased significantly in BIM attack, from 69% accuracy when using Lyapunov exponents, to 81% when the combination of both is employed.

To validate that the results from the combination (Lyapunov exponents and entropy) with respect to only Lyapunov exponents, an statistical test is performed to check whether there is a significant improvement. For this purpose, a two-sample *t*-test has been conducted to compare both Table 3 and Table 17. The data employed in this case are the performance values for the 7 methods × 12 attacks × 10 folds from cross validation, in each of the experiments, giving 840 pairs of data to perform the statistical evaluation. The results indicate that the null hypothesis is rejected, with a *p*-value of 0.0036. This means that both experiments reflect different means, indicating that the performance improvement exists and is statistically relevant.

Another aspect that is studied is the distribution of data and the presence of outliers, which could bias the results. As observed in Figure 10, some of the points in the Lyapunov exponents are far from the main distribution, which could lead to misinterpretations. However, most of them are in the same blob depending whether they belong to the adversarial set or not. For this purpose, an outlier detector test is performed, using the “median” method to highlight points of data that are further than three scaled median absolute deviations from the data distribution. This is performed for each Lyapunov exponent dimension, that are contained in different ranges of values. For the same experiments compared in the previous test, less than 2% of outliers are detected, so the data can be considered as meaningful enough to support the conclusions provided in this work.

In summary, the addition of Lyapunov exponents and entropy supposes and improvement in the classification for all the methods tested in this work. The good results of the combination can be explained in the following way. As stated in the introduction, entropy has been related to the information provided in the positive Lyapunov exponents, which in turn are the most discriminant to measure chaoticity. The addition of the entropy to the classifier, therefore, may have a boosting effect on these most discriminating first two exponents, leading to better overall performance.

## 4. Conclusions

In this paper we have studied the potential of a chaos theory framework in the problem of adversarial example classification. To study the chaotic (adversarial) inputs given to a neural network, two main approaches are considered. First, Maximal Lyapunov Exponents (MLE) have been tested as a suitable method on a wide range of conditions, such as with different adversarial attacks and using other sources of image noise that may cause the classification to fail. Then, based on a connection between entropy and Lyapunov exponents, we verified that the image processing transforms that reduced accuracy the most were also altering image entropy the most. More specifically, the experiments showed that there is a correlation between the image entropy variation and Lyapunov-based accuracy. Finally, while entropy alone was not useful for this problem, we showed that the combination of Lyapunov exponents and entropy produced better results than using either method alone. We explain this based on the relationship between entropy and positive Lyapunov exponents, which in fact are the most discriminant ones.

The exposed theoretical relationship between Lyapunov exponents and entropy, through the chaos theory translates into more powerful features to predict whether an image is adversarial. Moreover, this is achieved when adversarial perturbations are added, but also when entropy changing transformations fool the network.

The combination of both methods seems to support the results in a wider range of conditions, that could be applied to real world scenarios, where the noise of cameras, for example, can produce similar imperfections such as the ones covered in the different noise methods.

We suggest that future work should consider whether chaos theory concepts can be equally applied to the internal representation of the network (as opposed to the input image, which is the line followed in [10] and also in this work), as it has been initially proposed in [15]. The analogy of deep networks with chaotic systems (in which adversarial examples represent a tiny variation in the input that produces wildly different outputs) is very promising.

## Figures and Tables

**Table 1:** Comparison of different studies in the field.

Method	Datasets	Attacks	Processing Methods	Approach
Yin et al., 2019 [17]	MNIST, CIFAR10	PGD	None	Image space
Shumailov et al., 2020 [18]	MNIST	FGSM, PGD, BIM, CW	None	Layer activations
Vacanti et al., 2020 [19]	MNIST; FASHION, CIFAR10	CW, SPARSE, FGSM	None	Autoencoder
Freitas et al., 2020 [20]	PASCAL VOC + ImageNet	PGD, MI-FGSM	None	Feature alignment
Huang et al., 2019 [21]	MNIST, CIFAR10, ImageNet	BIM, DeepFool, CW	Gaussian	Statistical analysis
This work [2020]	MNIST, FASHION, CIFAR10	CW, FGSM, PGD, JSMA	EQ, CLAHE, Poisson	Lyapunov exponents
		DeepFool BIM, EAD, Virtual	S&P, Speckle, Gaussian	and entropy
		Madry, Spatial, HopSkip, Sparse		

**Table 2:** Attacks used and their parameters.

Attack	Parameters
CW	100 iterations; 0.1 learning rate; 10 tradeoff constant; $L_{2}$ norm
FGSM	0.3 epsilon; $L_{\infty }$ norm
PGD	0.3 epsilon; 10 iterations; $L_{\infty }$ norm
JSMA	1.0 theta; 1.0 gamma
DeepFool	0.02 overshoot; 50 iterations
BIM	0.3 epsilon; 10 iterations; $L_{\infty }$ norm
EAD	0.01 beta; 0.01 learning rate; 1000 iterations; 0.001 tradeoff constant
	FISTA regularization; 9 binary search steps; ElasticNet decision rule
Virtual	2.0 epsilon; 0.000001 xi; 1 iteration
Madry	0.3 epsilon; 40 iterations; 0.01 epsilon iteration; $L_{\infty }$ norm
Spatial	[−0.1,0.1] axis translation; [−30,30] angle rotation
HopSkip	100–10,000 number of evals; 64 iterations; geometric progression search
	1.0 gamma; $L_{2}$ norm
Sparse	10.0 epsilon; 20 iterations; 99% sparsity

**Table 3:** Accuracy of Lyapunov exponent-based adversarial example classification, showing robustness of the classification versus image transformation operations in the MNIST dataset.

	Base	EQ	CLAHE	Noise Poisson	Noise Salt and Pepper	Noise Speckle	Noise Localvar Gaussian
CW	100.00	54.71	84.85	71.81	99.38	100.00	100.00
DeepFool	99.47	91.74	97.50	98.36	99.38	98.75	100.00
FGM	95.26	91.02	96.73	100.00	95.79	96.87	100.00
JSMA	62.17	57.51	83.68	62.86	69.48	61.88	100.00
PGD	100.00	100.00	99.50	100.00	99.00	100.00	100.00
BIM	99.50	100.00	99.50	100.00	99.47	100.00	100.00
EAD	73.92	65.53	94.41	68.09	77.95	75.96	100.00
Virtual	100.00	58.19	94.17	100.00	100.00	100.00	100.00
Madry	100.00	100.00	99.00	100.00	99.50	100.00	100.00
Spatial	80.00	62.76	81.50	77.50	88.00	83.50	100.00
HopSkip	99.50	100.00	100.00	100.00	99.29	99.23	100.00
Sparse	63.14	66.24	87.56	100.00	81.29	68.86	100.00

**Table 4:** Accuracy of Lyapunov exponent-based adversarial example classification, showing robustness of the classification versus image transformation operations in the FASHION dataset.

	Base	EQ	CLAHE	Noise Poisson	Noise Salt and Pepper	Noise Speckle	Noise Localvar Gaussian
CW	95.84	75.26	86.11	50.62	99.44	96.54	96.01
DeepFool	84.92	91.08	80.53	75.78	84.04	80.50	97.14
FGM	90.58	90.53	93.71	99.50	92.13	92.71	99.47
JSMA	57.91	78.58	87.86	74.25	69.54	66.04	96.21
PGD	99.50	100.00	98.95	98.95	99.50	99.47	100.00
BIM	99.47	99.47	99.47	99.50	98.97	98.42	98.95
EAD	63.18	76.24	87.98	64.08	68.67	74.50	98.57
Virtual	95.13	91.33	96.92	98.33	91.22	95.90	98.33
Madry	99.47	100.00	98.95	98.97	98.95	99.47	99.50
Spatial	82.00	76.11	76.26	72.50	82.45	78.00	96.97
HopSkip	94.34	94.92	90.00	90.58	91.05	92.05	98.46
Sparse	55.88	74.71	81.81	98.71	74.25	66.54	99.33

**Table 5:** Accuracy of Lyapunov exponent-based adversarial example classification, showing robustness of the classification versus image transformation operations in the CIFAR dataset.

	Base	EQ	CLAHE	Noise Poisson	Noise Salt and Pepper	Noise Speckle	Noise Localvar Gaussian
CW	58.56	66.95	94.38	72.94	62.46	74.71	93.42
DeepFool	59.61	54.41	84.87	71.62	60.77	69.38	95.70
FGM	52.57	64.62	91.43	76.40	91.52	74.77	84.88
JSMA	51.58	54.54	88.82	62.35	69.64	76.70	92.40
PGD	82.92	88.77	93.63	90.38	96.32	87.66	93.65
BIM	72.52	79.22	93.24	86.54	91.73	83.10	89.84
EAD	50.49	54.71	85.33	66.51	64.52	74.12	89.15
Virtual	67.50	68.40	96.60	87.39	88.01	85.33	95.77
Madry	81.35	85.58	94.65	87.66	95.73	86.67	93.63
Spatial	98.97	96.42	96.95	97.39	97.47	97.95	65.11
HopSkip	47.78	55.48	89.12	68.13	72.12	80.15	90.26
Sparse	52.29	55.63	85.77	89.67	91.75	76.73	95.52

**Table 6:** $L_{0}$ metric for the different attacks in comparison with accuracy for Lyapunov exponents adversarial classification in the MNIST dataset.

Attack	*L_0_*	Lyap acc
CW	658.48	100.00
DeepFool	662.78	99.47
FGM	680.25	95.26
JSMA	18.33	62.17
PGD	783.88	100.00
BIM	765.03	99.50
EAD	109.52	73.92
Virtual	671.49	100.00
Madry	783.90	100.00
Spatial	333.51	80.00
HopSkip	632.13	99.50
Sparse	19.92	63.14

**Table 7:** Entropy of adversarial images with image processing methods in the MNIST dataset.

	Base	EQ	CLAHE	Noise Poisson	Noise Salt and Pepper	Noise Speckle	Noise Localvar Gaussian
test	1.53	1.53	2.24	1.60	1.61	1.78	5.92
CW	6.67	2.00	2.82	1.99	6.55	8.26	5.84
DeepFool	6.08	5.40	4.72	4.46	5.90	6.05	6.52
FGM	2.77	2.77	2.87	5.74	2.75	8.37	9.30
JSMA	1.60	1.60	2.30	1.68	1.65	1.87	5.97
PGD	6.28	6.24	4.10	7.45	6.03	9.37	9.37
BIM	4.31	4.28	3.43	6.19	4.20	9.28	9.35
EAD	2.56	2.47	2.77	2.37	2.58	2.68	6.19
Virtual	8.99	3.55	2.88	6.75	8.76	9.08	9.27
Madry	6.25	6.21	4.10	7.45	6.01	9.38	9.37
Spatial	3.58	3.13	3.15	2.89	3.55	3.54	6.32
HopSkip	8.19	7.48	5.96	6.04	7.94	8.11	6.65
Sparse	1.75	1.75	2.29	5.36	1.96	1.98	9.39

**Table 8:** Entropy of adversarial images with image processing methods in the FASHION dataset.

	Base	EQ	CLAHE	Noise Poisson	Noise Salt and Pepper	Noise Speckle	Noise Localvar Gaussian
test	4.03	4.03	3.91	4.02	4.00	5.07	7.34
CW	7.34	5.02	4.17	4.60	7.26	9.39	7.28
DeepFool	7.60	7.01	5.57	6.25	7.37	7.51	7.55
FGM	4.98	4.97	3.87	6.52	4.42	8.51	8.85
JSMA	4.01	4.01	3.88	4.06	3.95	5.10	7.33
PGD	7.42	7.38	4.69	7.51	6.78	9.05	9.02
BIM	6.18	6.11	4.26	7.05	5.61	8.95	8.98
EAD	5.19	5.10	4.44	4.94	5.08	5.79	7.47
Virtual	8.67	6.36	4.61	7.26	8.46	8.94	9.21
Madry	7.43	7.39	4.70	7.51	6.80	9.05	9.02
Spatial	5.90	5.33	4.40	4.81	5.76	5.86	7.28
HopSkip	8.82	7.87	6.21	6.81	8.55	8.71	7.57
Sparse	4.20	4.19	3.93	6.31	4.27	5.20	9.45

**Table 9:** Entropy of adversarial images with image processing methods in the CIFAR dataset.

	Base	EQ	CLAHE	Noise Poisson	Noise Salt and Pepper	Noise Speckle	Noise Localvar Gaussian
test	9.58	9.56	6.58	8.50	9.37	9.82	9.71
CW	9.90	9.83	6.63	8.61	9.67	9.83	9.67
DeepFool	9.95	9.90	6.60	8.58	9.72	9.83	9.71
FGM	9.68	9.65	5.96	7.99	8.85	9.31	9.28
JSMA	9.43	9.40	6.50	8.47	9.18	9.74	9.63
PGD	9.85	9.83	6.22	8.15	9.09	9.42	9.39
BIM	9.80	9.78	6.21	8.13	9.03	9.41	9.36
EAD	9.61	9.58	6.58	8.52	9.39	9.83	9.71
Virtual	10.00	9.99	6.66	8.29	9.68	9.82	9.81
Madry	9.85	9.83	6.24	8.16	9.08	9.44	9.39
Spatial	7.68	7.60	5.59	6.91	7.46	7.61	8.69
HopSkip	9.99	9.93	6.62	8.59	9.76	9.84	9.71
Sparse	9.59	9.58	6.56	8.02	9.37	9.82	9.81

**Table 10:** Entropy of data normalized with image processing.

	EQ	CLAHE	Noise Poisson	Noise Salt and Pepper	Noise Speckle	Noise Localvar Gaussian
CW	−0.61	−0.50	−0.61	−0.02	0.21	−0.11
DeepFool	−0.13	−0.26	−0.32	−0.03	−0.01	0.08
FGM	0.00	0.02	0.43	0.00	0.81	0.95
JSMA	0.00	0.12	0.01	0.01	0.05	0.75
PGD	−0.01	−0.39	0.21	−0.05	0.56	0.56
BIM	0.00	−0.14	0.30	−0.02	0.80	0.82
EAD	−0.01	0.04	−0.03	0.00	0.02	0.61
Virtual	−0.82	−0.92	−0.34	−0.03	0.01	0.04
Madry	−0.01	−0.39	0.22	−0.04	0.57	0.56
Spatial	−0.11	−0.11	−0.18	−0.01	−0.01	0.71

**Table 11:** Accuracy of entropy based adversarial example classification, showing robustness of the classification against image processing operations in the MNIST dataset.

	Base	EQ	CLAHE	Noise Poisson	Noise Salt and Pepper	Noise Speckle	Noise Localvar Gaussian
CW	99.50	61.50	72.00	60.00	99.50	100.00	57.00
DeepFool	100.00	98.50	99.50	96.50	100.00	100.00	82.00
FGM	97.50	97.50	88.00	98.50	99.00	100.00	100.00
JSMA	55.00	54.50	53.00	55.50	52.50	55.00	44.00
PGD	100.00	100.00	100.00	100.00	100.00	100.00	100.00
BIM	100.00	100.00	99.50	100.00	100.00	100.00	100.00
EAD	89.50	86.00	80.00	80.50	87.00	82.00	71.50
Virtual	97.50	63.00	63.00	97.50	97.50	97.50	97.50
Madry	100.00	100.00	100.00	100.00	100.00	100.00	100.00
Spatial	94.00	93.00	80.00	89.00	94.00	92.50	77.50
HopSkip	100.00	100.00	100.00	100.00	100.00	100.00	91.00
Sparse	63.50	63.00	53.50	99.50	70.50	57.50	99.50

**Table 12:** Accuracy of entropy based adversarial example classification, showing robustness of the classification against image processing operations in the FASHION dataset.

	Base	EQ	CLAHE	Noise Poisson	Noise Salt and Pepper	Noise Speckle	Noise Localvar Gaussian
CW	92.00	58.00	53.50	56.00	92.50	95.00	51.50
DeepFool	96.00	91.00	87.50	83.00	95.50	81.00	54.50
FGM	59.50	60.00	51.50	93.00	53.50	91.00	81.50
JSMA	44.00	36.50	37.50	44.00	45.50	40.00	46.00
PGD	96.50	96.50	73.00	96.50	95.50	94.50	89.00
BIM	89.00	88.50	54.50	94.50	82.50	93.50	87.00
EAD	65.50	64.00	58.00	60.50	66.00	55.00	53.50
Virtual	95.50	69.00	60.00	90.50	95.00	89.50	91.50
Madry	96.50	96.50	74.50	96.50	95.50	94.00	88.50
Spatial	75.50	64.00	58.50	59.00	74.00	55.00	52.50
HopSkip	100.00	97.50	96.50	92.00	100.00	93.00	52.50
Sparse	52.50	52.00	44.50	89.50	53.50	50.00	95.50

**Table 13:** Accuracy of entropy based adversarial example classification, showing robustness of the classification against image processing operations in the CIFAR dataset.

	Base	EQ	CLAHE	Noise Poisson	Noise Salt and Pepper	Noise Speckle	Noise Localvar Gaussian
CW	59.50	58.00	55.50	53.00	59.00	45.50	47.50
DeepFool	61.00	60.00	46.50	51.50	61.50	43.50	44.00
FGM	49.50	52.00	81.00	66.50	65.50	69.50	65.00
JSMA	54.50	55.50	57.50	52.00	56.50	54.00	55.00
PGD	56.50	55.50	76.50	63.50	62.00	67.50	64.00
BIM	53.50	53.50	76.50	63.50	62.00	68.50	65.00
EAD	48.50	45.50	46.00	43.00	47.00	46.50	46.50
Virtual	63.50	64.00	57.50	68.00	60.50	48.00	57.50
Madry	56.50	56.00	74.00	64.00	63.00	68.00	64.00
Spatial	96.00	96.00	97.50	95.50	96.00	98.50	95.50
HopSkip	63.00	60.00	51.00	53.00	63.00	51.00	49.50
Sparse	46.00	50.00	47.50	71.00	45.50	52.00	55.50

**Table 14:** Accuracy comparison between using entropy or Lyapunov in adversarial example classification, showing robustness of the classification against image processing operations in the MNIST dataset.

	Base	EQ	CLAHE	Noise Poisson	Noise Salt and Pepper	Noise Speckle	Noise Localvar Gaussian
CW	−0.50	**6.79**	−12.85	−11.81	**0.12**	0.00	−43.00
DeepFool	**0.53**	**6.76**	**2.00**	−1.86	**0.63**	**1.25**	−18.00
FGM	**2.24**	**6.48**	−8.73	−1.50	**3.21**	**3.13**	0.00
JSMA	−7.17	−3.01	−30.68	−7.36	−16.98	−6.88	−56.00
PGD	0.00	0.00	**0.50**	0.00	**1.00**	0.00	0.00
BIM	**0.50**	0.00	0.00	0.00	**0.53**	0.00	0.00
EAD	**15.58**	**20.47**	−14.41	**12.41**	**9.05**	**6.04**	−28.50
Virtual	−2.50	**4.81**	−31.17	−2.50	−2.50	−2.50	−2.50
Madry	0.00	0.00	**1.00**	0.00	**0.50**	0.00	0.00
Spatial	**14.00**	**30.24**	−1.50	**11.50**	**6.00**	**9.00**	−22.50
HopSkip	**0.50**	0.00	0.00	0.00	**0.71**	**0.77**	−9.00
Sparse	**0.36**	−3.24	−34.06	−0.50	−10.79	−11.36	−0.50

**Table 15:** Accuracy comparison between using entropy or Lyapunov in adversarial example classification, showing robustness of the classification against image processing operations in the FASHION dataset.

	Base	EQ	CLAHE	Noise Poisson	Noise Salt and Pepper	Noise Speckle	Noise Localvar Gaussian
CW	−3.84	−17.26	−32.61	**5.38**	−6.94	−1.54	−44.51
DeepFool	**11.08**	−0.08	**6.97**	**7.22**	**11.46**	**0.50**	−42.64
FGM	−31.08	−30.53	−42.21	−6.50	−38.63	−1.71	−17.97
JSMA	−13.91	−42.08	−50.36	−30.25	−24.04	−26.04	−50.21
PGD	−3.00	−3.50	−25.95	−2.45	−4.00	−4.97	−11.00
BIM	−10.47	−10.97	−44.97	−5.00	−16.47	−4.92	−11.95
EAD	**2.32**	−12.24	−29.98	−3.58	−2.67	−19.50	−45.07
Virtual	**0.37**	−22.33	−36.92	−7.83	**3.78**	−6.40	−6.83
Madry	−2.97	−3.50	−24.45	−2.47	−3.45	−5.47	−11.00
Spatial	−6.50	−12.11	−17.76	−13.50	−8.45	−23.00	−44.47
HopSkip	**5.66**	**2.58**	**6.50**	**1.42**	**8.95**	**0.95**	−45.96
Sparse	−3.38	−22.71	−37.31	−9.21	−20.75	−16.54	−3.83

**Table 16:** Accuracy comparison between using entropy or Lyapunov in adversarial example classification, showing robustness of the classification against image processing operations in the CIFAR dataset.

	Base	EQ	CLAHE	Noise Poisson	Noise Salt and Pepper	Noise Speckle	Noise Localvar Gaussian
CW	**0.94**	−8.95	−38.88	−19.94	−3.46	−29.21	−45.92
DeepFool	**1.39**	**5.59**	−38.37	−20.12	**0.73**	−25.88	−51.70
FGM	−3.07	−12.62	−10.43	−9.90	−26.02	−5.27	−19.88
JSMA	**2.92**	**0.96**	−31.32	−10.35	−13.14	−22.70	−37.40
PGD	−26.42	−33.27	−17.13	−26.88	−34.32	−20.16	−29.65
BIM	−19.02	−25.72	−16.74	−23.04	−29.73	−14.60	-24.84
EAD	−1.99	−9.21	−39.33	−23.51	−17.52	−27.62	−42.65
Virtual	−4.00	−4.40	−39.10	−19.39	−27.51	−37.33	−38.27
Madry	−24.85	−29.58	−20.65	−23.66	−32.73	−18.67	−29.63
Spatial	−2.97	−0.42	**0.55**	−1.89	−1.47	**0.55**	**30.39**
HopSkip	**15.22**	**4.52**	−38.12	−15.13	−9.12	−29.15	−40.76
Sparse	−6.29	−5.63	−38.27	−18.67	−46.25	−24.73	−40.02

**Table 17:** Accuracy using both Lyapunov and entropy in adversarial example classification, showing robustness of the classification against image processing operations in the MNIST dataset.

	Base	EQ	CLAHE	Noise Poisson	Noise Salt and Pepper	Noise Speckle	Noise Localvar Gaussian
CW	100.00	86.09	79.04	81.11	100.00	100.00	56.93
DeepFool	100.00	96.27	100.00	100.00	100.00	100.00	93.45
FGM	100.00	96.09	88.95	100.00	99.47	100.00	100.00
JSMA	66.00	85.18	70.19	66.71	67.14	68.38	67.38
PGD	100.00	100.00	100.00	100.00	100.00	100.00	100.00
BIM	100.00	100.00	100.00	100.00	100.00	100.00	100.00
EAD	87.26	84.78	81.54	81.80	86.10	86.88	84.39
Virtual	100.00	84.89	97.18	100.00	100.00	100.00	100.00
Madry	100.00	100.00	99.44	100.00	100.00	100.00	100.00
Spatial	99.00	99.09	83.13	94.95	95.00	97.97	87.84
HopSkip	100.00	100.00	100.00	100.00	100.00	100.00	95.45
Sparse	65.81	85.83	80.61	100.00	78.43	72.38	100.00

**Table 18:** Accuracy using both Lyapunov and entropy in adversarial example classification, showing robustness of the classification against image processing operations in the FASHION dataset.

	Base	EQ	CLAHE	Noise Poisson	Noise Salt and Pepper	Noise Speckle	Noise Localvar Gaussian
CW	97.81	75.05	50.79	59.60	100.00	100.00	56.58
DeepFool	95.58	86.90	85.42	91.54	96.43	92.24	66.22
FGM	90.56	88.90	90.10	98.33	90.20	96.67	93.76
JSMA	55.38	73.18	63.59	61.90	54.78	59.90	58.19
PGD	100.00	98.67	92.48	99.44	99.44	99.44	92.71
BIM	99.44	92.52	88.53	98.86	98.86	97.75	92.12
EAD	75.64	73.21	63.60	70.21	69.78	73.86	68.85
Virtual	99.09	92.09	91.97	99.09	99.09	96.36	97.27
Madry	100.00	98.67	91.99	98.33	98.82	98.89	93.82
Spatial	93.13	79.29	78.79	76.64	88.39	84.09	60.64
HopSkip	100.00	95.95	98.24	97.35	100.00	98.26	77.20
Sparse	60.42	58.11	65.13	98.62	60.86	63.00	97.86

**Table 19:** Accuracy using both Lyapunov and entropy in adversarial example classification, showing robustness of the classification against image processing operations in the CIFAR dataset.

	Base	EQ	CLAHE	Noise Poisson	Noise Salt and Pepper	Noise Speckle	Noise Localvar Gaussian
CW	61.79	65.61	71.44	50.38	61.29	57.42	57.12
DeepFool	62.44	58.48	68.79	53.27	61.18	49.85	56.14
FGM	62.20	74.73	90.13	83.85	86.73	77.69	81.98
JSMA	62.14	65.98	66.45	54.95	70.00	60.19	64.62
PGD	85.38	91.65	89.36	86.26	95.38	73.35	70.58
BIM	78.01	81.35	86.82	82.76	86.67	76.35	72.18
EAD	59.49	56.21	68.48	53.33	63.91	57.80	52.58
Virtual	79.09	78.03	88.71	75.00	76.28	54.32	62.73
Madry	84.45	90.88	89.36	84.40	95.26	79.18	66.92
Spatial	98.62	98.57	99.23	98.67	98.52	98.57	97.14
HopSkip	62.86	59.70	69.68	45.96	65.08	34.70	63.45
Sparse	49.12	41.14	64.82	80.82	84.77	55.90	65.53
